# Microaxial Flow Pump Support as a Bridge to Transaxillary TAVI‐In‐TAVI in Cardiogenic Shock

**DOI:** 10.1002/ccr3.73225

**Published:** 2026-07-22

**Authors:** Matteo Orlandi, Niccolò Ciardetti, Lucrezia Biagiotti, Maria Federica Crociani, Giulio Grandi, Miroslava Stolcova, Francesca Ristalli, Carlo Di Mario, Alessio Mattesini, Francesco Meucci

**Affiliations:** ^1^ Department of Experimental and Clinical Medicine University of Florence Florence Italy; ^2^ Division of Structural Interventional Cardiology, Cardiothoracovascular Department Careggi University Hospital Florence Italy

**Keywords:** bioprosthesis failure, cardiogenic shock, mechanical circulatory support, percutaneous coronary intervention, transcatheter aortic valve replacement

## Abstract

A 79‐year‐old man presented with cardiogenic shock due to severe degeneration of a prior transcatheter aortic valve. Temporary mechanical circulatory support with a transvalvular microaxial flow pump was initiated, resulting in rapid stabilization. A staged transaxillary TAVI‐in‐TAVI procedure was subsequently performed with a favorable outcome.

## Key Clinical Message

Bioprosthetic valve dysfunction after transcatheter aortic valve implantation may present with cardiogenic shock. In high‐risk patients, temporary mechanical circulatory support can stabilize hemodynamics and allow a staged transcatheter valve‐in‐valve procedure, offering a feasible and effective treatment strategy when immediate intervention carries excessive procedural risk.

## 1 Introduction

Transcatheter aortic valve implantation (TAVI) has transformed the management of severe aortic stenosis (AS), expanding from high‐risk to intermediate‐ and low‐risk populations [1, 2]. As indications extend to younger patients, long‐term durability and lifetime management of transcatheter heart valves have become increasingly important. All bioprosthetic valves are subject to bioprosthetic valve dysfunction (BVD) [3]. Although mid‐term outcomes are reassuring, with low rates of clinically relevant structural valve deterioration at 5–8 years, long‐term durability data remain limited, and redo procedures such as TAVI‐in‐TAVI are expected to increase [4, 5].

BVD may lead to acute hemodynamic deterioration and can precipitate cardiogenic shock, a condition defined as sustained tissue hypoperfusion due to cardiac dysfunction [6] and associated with high mortality despite advances in treatment [7]. In this setting, temporary mechanical circulatory support (MCS) may provide a bridge to definitive therapy [8]. Although the use of percutaneous microaxial flow pumps in patients with severe aortic valve disease has historically been considered challenging, increasing experience supports their feasibility in selected high‐risk patients [9]. However, evidence in patients with cardiogenic shock due to degenerated transcatheter bioprostheses, particularly in the context of TAVI‐in‐TAVI, remains limited.

## 2 Case Presentation

### 2.1 Case History

In May 2025, a 79‐year‐old man presented to the emergency department with sudden‐onset dyspnea and chest pain. On arrival, he was tachycardic (120 bpm), tachypneic, and hypotensive (blood pressure 70/30 mmHg), with clinical signs of systemic hypoperfusion, including cold extremities and altered mental status. He was breathing spontaneously but required low‐flow oxygen supplementation via nasal cannulae. Initial point‐of‐care testing revealed type 1 respiratory failure and elevated lactate levels (3 mmol/L). The presenting electrocardiogram (ECG) showed sinus tachycardia with diffuse ST‐segment depression and ST‐segment elevation in aVR, raising concern for extensive myocardial ischemia. In the setting of suspected cardiogenic shock with high‐risk ECG features, the patient was emergently transferred to the catheterization laboratory.

The patient had several cardiovascular risk factors (i.e., type 2 diabetes mellitus, dyslipidemia, former smoker) and a complex cardiovascular history with multiple comorbidities. He had longstanding severe bilateral peripheral artery disease, predominantly affecting the left femoro‐popliteal axis, requiring multiple percutaneous transluminal angioplasty procedures. His medical history was also notable for right lung cancer treated with pneumonectomy, bladder cancer treated with transurethral resection, and a relapse of lung cancer involving the left lung, managed with radiotherapy 1 year earlier. From a cardiovascular standpoint, in 2019 he developed severe symptomatic AS associated with chronic coronary syndrome. He underwent TAVI with a self‐expandable 26 mm Evolut R bioprosthesis (Medtronic, Minneapolis, MN, USA) and concomitant percutaneous coronary intervention (PCI) with stent implantation to the mid left anterior descending artery. Two months before presentation, the patient was admitted for acute heart failure. Transthoracic echocardiography demonstrated preserved left ventricular ejection fraction (LVEF, 52%) and severe aortic BVD, with a mean transvalvular gradient of 50 mmHg, consistent with advanced structural valve deterioration. Coronary angiography revealed a 90% ostial stenosis of the first diagonal branch, which was treated with PCI. One month later, he was readmitted for exertional chest pain and underwent a complete thoraco‐abdominal computed tomography (CT) angiography. Given progressive BVD and prohibitive surgical risk, the Heart Team recommended elective transaxillary TAVI‐in‐TAVI. He was subsequently discharged home; however, before the planned procedure, he presented to the emergency department with acute clinical deterioration.

### 2.2 Differential Diagnosis

At presentation, the leading diagnostic consideration was acute coronary syndrome complicated by cardiogenic shock, given the ischemic ECG pattern. However, known aortic BVD was also considered, potentially resulting in severe transvalvular obstruction or acute regurgitation and contributing to hemodynamic collapse. A mixed ischemic–valvular mechanism of cardiogenic shock was therefore suspected. Mechanical complications of myocardial infarction were considered less likely in the absence of suggestive clinical or ECG findings.

### 2.3 Investigations

Urgent bedside transthoracic echocardiography revealed severe diffuse hypokinesia of the left ventricle with an estimated LVEF of approximately 10% in the absence of acute mechanical complications. The bioprosthetic valve showed severe stenotic dysfunction without regurgitation.

In the catheterization laboratory, an ultrasound‐guided right femoral access was obtained, using a 45‐cm sheath, and angiography confirmed severe calcific peripheral vascular disease with critical stenosis of the right common iliac artery and of the left external iliac artery. Coronary angiography demonstrated a dominant left circumflex artery with a critical proximal stenosis and haziness, a long calcific lesion of the left anterior descending artery, and noncritical disease of the remaining vessels.

### 2.4 Treatment

An urgent PCI of the left circumflex artery was performed. Lesion preparation included predilation with a 2.5‐mm noncompliant balloon, followed by treatment with a 2.5 × 15 mm paclitaxel‐eluting balloon (Prevail; Medtronic, Minneapolis, MN, USA). Due to a suboptimal angiographic result, a 2.5 × 15 mm everolimus‐eluting stent (Xience; Abbott Vascular, Santa Clara, CA, USA) was implanted, achieving optimal angiographic result with TIMI 3 flow.

Despite successful coronary revascularization, the patient remained hemodynamically unstable, with persistent hypotension and hypoperfusion requiring escalating vasoactive support. The absence of immediate hemodynamic improvement suggested a predominant valvular contribution to shock. After a multidisciplinary discussion involving interventional cardiologists and intensive care specialists, MCS with a transvalvular microaxial flow pump was initiated during the same procedure. Although the presence of a bioprosthetic aortic valve represents a relative contraindication, this strategy was favored due to its ability to provide immediate forward flow and left ventricular unloading. To facilitate large‐bore device passage through the severely calcified iliac axis, intravascular lithotripsy was performed using a 7.0 mm Shockwave M5+ balloon (Shockwave Medical, Santa Clara, CA, USA). The degenerated bioprosthetic valve was crossed with an Amplatz left 1 diagnostic catheter and a straight stiff guidewire. Invasive trans‐prosthetic hemodynamic assessment demonstrated a peak‐to‐peak gradient of 30 mmHg, confirming clinically relevant valve obstruction. A percutaneous microaxial flow pump (Impella CP; Abiomed, Danvers, MA, USA) was then advanced across the bioprosthetic valve and positioned in the left ventricle. Device implantation resulted in immediate hemodynamic stabilization, allowing transfer to the intensive care unit. The key procedural steps, including femoral access, coronary angiography, PCI and percutaneous microaxial flow pump positioning, are illustrated in Figure 1.

**FIGURE 1:**
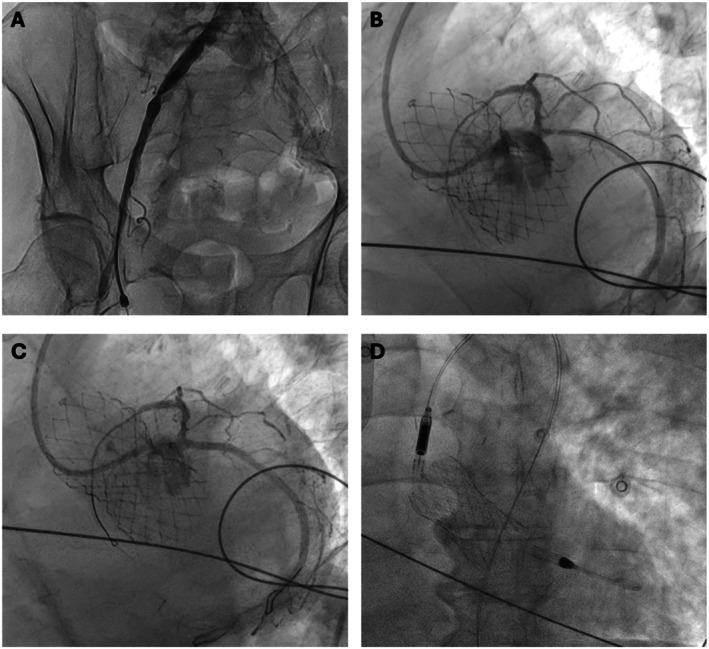
Key procedural steps during initial management (A) Angiography showing critical stenosis of the right common iliac artery. (B) Left coronary angiography demonstrating critical proximal left circumflex artery stenosis and noncritical disease of the left anterior descending artery. (C) Final angiographic result after percutaneous coronary intervention of the left circumflex artery. (D) Positioning of the percutaneous microaxial flow pump in the left ventricle.

The stabilization phase was used to coordinate a definitive valve intervention. Given the presence of severe peripheral vascular disease and hostile femoral access, a staged transaxillary TAVI‐in‐TAVI strategy was selected. CT images were analyzed to guide procedural planning for TAVI‐in‐TAVI. Based on transcatheter heart valve area measurements, implantation of an overfilled 23 mm balloon‐expandable transcatheter heart valve (Edwards SAPIEN 3 Ultra; Edwards Lifesciences, Irvine, CA, USA) was planned. The coronary risk plane, corresponding to the inferior margin of the left coronary artery ostium, was identified at node 5. Additional CT parameters were assessed to estimate the risk of coronary obstruction, including virtual transcatheter valve‐to‐coronary (VTC) and virtual transcatheter valve‐to‐aortic‐sinus (VTAoS) distances (right VTC 4.9 mm, left VTC 4.1 mm; right VTAoS 2.3 mm, whereas the left VTAoS was not measurable because of direct apposition between the virtual transcatheter valve frame and the aortic sinus wall). Despite the minimal left VTAoS, the risk of coronary obstruction was considered acceptable provided that the neoskirt plane, corresponding to the outflow edge of the second prosthesis, did not extend above the coronary risk plane [10]. Therefore, neither leaflet modification techniques nor prophylactic coronary protection strategies were performed.

Following approximately 18 h of hemodynamic stabilization under microaxial flow pump support, the patient was brought back to the catheterization laboratory for definitive transaxillary TAVI‐in‐TAVI. Left axillary arterial access was obtained under ultrasound guidance, with two pre‐implanted Perclose ProStyle closure devices (Abbott Vascular, Santa Clara, CA, USA), and a 14‐F sheath was inserted. In parallel, an echo‐ and angio‐guided left femoral arterial access was obtained using a 6‐F sheath for ancillary catheter support. Before removal of the microaxial flow pump, the degenerated transcatheter valve was crossed with an Amplatz left 1 diagnostic catheter and a straight stiff guidewire. After removal of the microaxial flow pump, a Safari2 XS guidewire (Boston Scientific, Marlborough, MA, USA) was positioned in the left ventricle, and an overfilled (nominal volume plus 3 mL) 23 mm balloon‐expandable transcatheter heart valve (Edwards SAPIEN 3 Ultra; Edwards Lifesciences, Irvine, CA, USA) was immediately advanced and successfully implanted within the degenerated transcatheter valve during rapid left ventricular pacing. Final angiography confirmed correct valve positioning with no residual regurgitation. Despite positioning of the outflow edge of the second transcatheter heart valve at node 6, above the coronary risk plane, final angiography demonstrated preserved patency of the left and right coronary arteries. Hemostasis of the axillary access site was reinforced using balloon‐assisted techniques. The transaxillary TAVI‐in‐TAVI procedure is illustrated in Figure 2.

**FIGURE 2:**
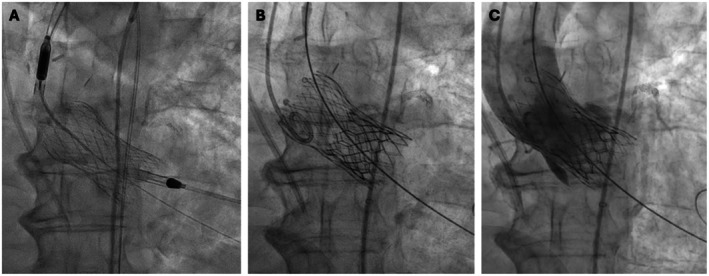
Transaxillary TAVI‐in‐TAVI procedure. (A) Crossing of the degenerated transcatheter heart valve with an Amplatz Left 1 diagnostic catheter and a straight stiff guidewire. (B) Implantation of a balloon‐expandable transcatheter heart valve within the degenerated prosthesis. (C) Final angiographic result demonstrating optimal valve positioning, patent coronary arteries, and no residual regurgitation; the outflow portion of the balloon‐expandable prosthesis is aligned approximately at node 6 of the underlying self‐expanding valve.

On the following days, hemodynamics improved, and inotropic and vasoactive support was gradually weaned. From a respiratory standpoint, noninvasive ventilation was initially required and subsequently discontinued, with transition to low‐flow oxygen via nasal cannula. Diuresis improved with progressively reduced doses of furosemide. During the following days, the patient was able to mobilize and ambulate.

### 2.5 Outcomes and Follow‐Up

A pre‐discharge transthoracic echocardiogram demonstrated recovery of left ventricular systolic function, with an LVEF of approximately 50%. The transcatheter aortic valve prosthesis was well positioned, with normal antegrade gradients and no intravalvular or paravalvular regurgitation. The post‐procedural echocardiographic result is shown in Figure 3. On postoperative day 8, the patient was discharged to a cardiac rehabilitation center. At discharge, the patient was hemodynamically stable, with a blood pressure of 95/45 mmHg and a heart rate of 91 bpm. Oxygen saturation was 99% while receiving supplemental oxygen through low‐flow nasal cannulae with a fraction of inspired oxygen of 28%. At 12‐month follow‐up, the patient was alive and clinically stable, in New York Heart Association functional class II and without angina. Echocardiographic findings were unchanged compared with discharge. A CT angiography confirmed the optimal result of the procedure, with appropriate valve positioning and no complications (Figure 4).

**FIGURE 3:**
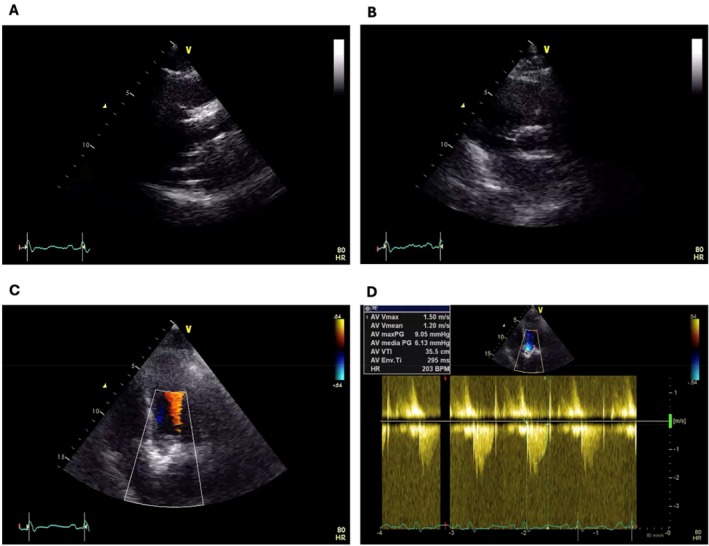
Post‐procedural echocardiographic assessment. (A) Parasternal long‐axis view. (B) Parasternal short‐axis view. (C) Apical five‐chamber view with color Doppler demonstrating no residual regurgitation. (D) Continuous‐wave Doppler showing a mean transvalvular gradient of 6.1 mmHg.

**FIGURE 4:**
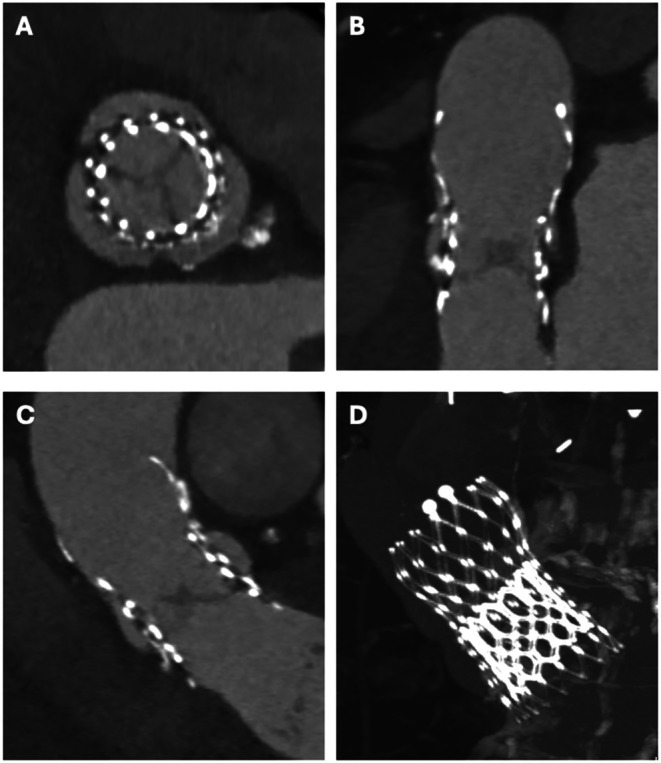
Computed tomography angiography at 6‐month follow‐up. (A) Transverse view. (B) Sagittal view. (C) Coronal view. (D) Coronal‐oblique view. CT angiography confirmed the optimal result of the TAVI‐in‐TAVI procedure, with the outflow edge of the balloon‐expandable transcatheter heart valve positioned at node 6 of the underlying self‐expanding prosthesis, appropriate valve expansion, and no evidence of device‐related complications.

## 3 Discussion

This case highlights the complexity of managing cardiogenic shock in patients with degenerated transcatheter bioprostheses and multiple comorbidities. In this setting, rapid identification of the predominant mechanism of shock and timely intervention are critical. Following successful coronary revascularization, continuous reassessment by a multidisciplinary Heart Team involving interventional cardiologists and intensive care specialists, led to the recognition of the predominant valvular contribution to shock. This supported the decision to initiate temporary MCS and subsequently proceed with a staged transaxillary TAVI‐in‐TAVI strategy.

Immediate definitive valve intervention was considered prohibitively high risk because of multiple factors: anticipated severe aortic regurgitation during valve manipulation, the need for rapid ventricular pacing in a patient with minimal hemodynamic reserve, hostile femoral access, and the absence of immediate transcatheter heart valve availability.

In this context, temporary MCS played a pivotal role, enabling rapid hemodynamic stabilization and preservation of end‐organ perfusion. By augmenting cardiac output and mean arterial pressure while unloading the left ventricle, [11] microaxial flow pump support allowed safe bridging to definitive treatment. Although severe aortic valve obstruction has historically been considered a relative contraindication to microaxial flow pump support, some early experience has demonstrated the feasibility of device implantation across stenotic native and bioprosthetic valves in selected patients, even if data remain limited [9, 12]. In the present case, the need for rapid restoration of systemic perfusion and left ventricular unloading outweighed the potential procedural risks, making temporary MCS a valuable bridge to definitive valve treatment. The choice of a microaxial flow pump over an intra‐aortic balloon pump was driven by the underlying shock mechanism. In the presence of severe bioprosthetic valve obstruction, the goal was not only circulatory support but also active transvalvular flow generation. By directly transferring blood from the left ventricle to the ascending aorta, the microaxial flow pump functionally overcame the obstructive valve physiology and provided a bridge to definitive TAVI‐in‐TAVI.

A key element in this case was the availability of preprocedural CT imaging, which allowed rapid procedural planning without the need for additional imaging in the acute setting. In a patient with cardiogenic shock, obtaining high‐quality CT imaging would have been impractical and potentially unsafe; therefore, prior imaging was instrumental in assessing vascular access, valve anatomy, and the risk of coronary obstruction, enabling timely execution of a TAVI‐in‐TAVI strategy.

An additional noteworthy finding was the absence of coronary obstruction despite final positioning of the outflow edge of the balloon‐expandable valve at node 6, above the pre‐defined coronary risk plane. Coronary obstruction remains one of the major concerns during TAVI‐in‐TAVI procedures, particularly in short‐in‐tall combinations. Recent fluoroscopic analyses of SAPIEN 3 Ultra implantation within failed self‐expanding prostheses demonstrated upward valve movement in nearly half of cases, highlighting the potential for a higher‐than‐anticipated final valve position and consequent coronary compromise [13]. In the present case, despite the high final position of the second prosthesis and a minimal left VTAoS, both coronary arteries remained patent at final angiography and follow‐up imaging. A possible explanation is partial recoil of the balloon‐expandable valve frame, which may have resulted in a lower effective neoskirt than anticipated from angiographic assessment alone. This, in turn, may have preserved a residual space between the two valve frames at the level of the coronary ostia, allowing continued coronary blood flow despite the high final valve position.

The choice of a transaxillary approach also deserves consideration. Current European and American guidelines identify transfemoral access as the preferred route for TAVI whenever feasible [1, 2]. However, in the present case, severe peripheral artery disease precluded a transfemoral strategy, while the patient's extreme surgical risk rendered surgical valve replacement unsuitable. Although specific recommendations for access selection in TAVI‐in‐TAVI procedures are lacking, this case illustrates that transaxillary access may represent a valuable alternative in carefully selected patients with hostile iliofemoral anatomy.

## 4 Conclusions

This case demonstrates that in carefully selected high‐risk patients with BVD, a staged, heart team‐guided approach with MCS followed by definitive treatment of the underlying valvular disease may allow hemodynamic stabilization and favorable clinical outcomes despite extreme procedural complexity.

## Author Contributions

**Niccolò Ciardetti:** writing – original draft. **Alessio Mattesini:** conceptualization, writing – review and editing. **Francesco Meucci:** writing – review and editing, conceptualization, supervision. **Miroslava Stolcova:** writing – review and editing. **Matteo Orlandi:** writing – original draft, visualization. **Giulio Grandi:** writing – review and editing. **Francesca Ristalli:** writing – review and editing. **Lucrezia Biagiotti:** writing – review and editing. **Maria Federica Crociani:** writing – review and editing. **Carlo Di Mario:** conceptualization, supervision.

## Funding

The authors have nothing to report.

## Ethics Statement

Ethical approval was not required for this case report in accordance with local and institutional policies.

## Consent

Written informed consent was obtained from the patient for publication of this case report and any accompanying images, in accordance with the journal's patient consent policy.

## Conflicts of Interest

The authors declare no conflicts of interest.

## Data Availability

The data that support the findings of this study are available on request from the corresponding author. The data are not publicly available due to privacy or ethical restrictions.
